# A Simple, Non-Invasive Score to Predict Paroxysmal Atrial Fibrillation

**DOI:** 10.1371/journal.pone.0163621

**Published:** 2016-09-28

**Authors:** Stefan M. Kallenberger, Christian Schmid, Felix Wiedmann, Derliz Mereles, Hugo A. Katus, Dierk Thomas, Constanze Schmidt

**Affiliations:** 1 Department for Bioinformatics and Functional Genomics, Division of Theoretical Bioinformatics, German Cancer Research Center (DKFZ), Heidelberg, Germany; 2 Institute for Pharmacy and Molecular Biotechnology (IPMB) and BioQuant, Heidelberg University, Heidelberg, Germany; 3 Department of Cardiology, University of Heidelberg, Heidelberg, Germany; 4 German Centre for Cardiovascular Research (DZHK), partner site Heidelberg/Mannheim, University of Heidelberg, Heidelberg, Germany; University at Buffalo—The State University of New York, UNITED STATES

## Abstract

Paroxysmal atrial fibrillation (pAF) is a major risk factor for stroke but remains often unobserved. To predict the presence of pAF, we developed model scores based on echocardiographic and other clinical parameters from routine cardiac assessment. The scores can be easily implemented to clinical practice and might improve the early detection of pAF. In total, 47 echocardiographic and other clinical parameters were collected from 1000 patients with sinus rhythm (SR; n = 728), pAF (n = 161) and cAF (n = 111). We developed logistic models for classifying between pAF and SR that were reduced to the most predictive parameters. To facilitate clinical implementation, linear scores were derived. To study the pathophysiological progression to cAF, we analogously developed models for cAF prediction. For classification between pAF and SR, amongst 12 selected model parameters, the most predictive variables were tissue Doppler imaging velocity during atrial contraction (TDI, A’), left atrial diameter, age and aortic root diameter. Models for classifying between pAF and SR or between cAF and SR showed areas under the ROC curves of 0.80 or 0.93, which resembles classifiers with high discriminative power. The novel risk scores were suitable to predict the presence of pAF based on variables readily available from routine cardiac assessment. Modelling helped to quantitatively characterize the pathophysiologic transition from SR via pAF to cAF. Applying the scores may improve the early detection of pAF and might be used as decision aid for initiating preventive interventions to reduce AF-associated complications.

## Introduction

Atrial fibrillation (AF) is the most frequent rhythm disorder, and its prevalence is expected to further increase due to demographic transition [1]. In some cases, AF is firstly diagnosed after stroke or a transient ischemic event. For this reason, early diagnosis of AF episodes is essential. In particular, paroxysmal AF (pAF) remains often unobserved, in contrast to chronic AF (cAF), and is a frequent cause of cryptogenic ischemic stroke [2–5]. Further optimization of easy implementable non-invasive methods for pAF detection represents an important task for translational electrophysiological research, as recently declared in the EHRA roadmap to improve the quality of atrial fibrillation management [4].

Traditionally, surface electrocardiogram (ECG) is the basic method for AF diagnosis. Holter ECG monitoring is used to detect pAF [6]. In addition, intra-cardiac ECG measured with cardiac device electrodes or catheter electrodes during ablation procedures is used for AF detection. Risk stratification tools were established for the prevention of stroke, transient ischemic attacks or other thromboembolic complications. In particular, the CHADS_2_ and CHA_2_DS_2_-VASc scores are part of the common clinical practice for guiding prophylactic anticoagulation therapy [6]. It can be expected that, within the context of the evolving area of systems medicine, further predictive models will be developed, which integrate clinical parameters from different diagnostic techniques, to predict the individual risk for the development of pathologies and can be used to optimize personalized therapies.

Previous studies analyzed the pathophysiological involvement of echocardiographic parameters that reflect hemodynamic alterations in the development of AF, in order to improve the risk assessment of individual patients for developing AF. Patients with non-rheumatic atrial fibrillation showed left atrial (LA) enlargement, increased left ventricular (LV) wall thickness, and reduced end-diastolic to end-systolic fractional shortening of the LV [7]. It was shown that at higher age, echocardiographic measures of the diastolic function are significantly associated with an increased risk of AF [8]. Left ventricular dysfunction and LA size were shown to be predictive for thromboembolic events in patients with non-valvular AF [9].

While cAF can be easily detected, pAF remains often unobserved. In this study, we therefore focused on the detection of pAF. We combined in total 47 echocardiographic parameters and other clinical parameters to develop a predictive model score for the presence of pAF. To study pathophysiological aspects of changes of echocardiographic parameters in AF, we developed in a similar manner models for classification between sinus rhythm (SR) and cAF. In the clinical practice, a model score for pAF prediction might contribute to the early detection of pAF in patients undergoing an echocardiographic investigation, and therefore creates an additional diagnostic value of echocardiographic parameters. The indication of a risk for pAF could suggest conducting further electrophysiological investigations to verify the presence of pAF.

## Methods

### Study population

Echocardiographic and additional clinical data of 1000 patients were collected between January 2009 and July 2015 at the Department of Cardiology of the University Hospital of Heidelberg (Germany). Patient data were included in this retrospective study in a de-identified manner and classified to the groups SR, pAF or cAF. The category cAF subsumed the possible subcategories persistent, long-standing and permanent AF [6]. AF stages were taken from patient histories. Within the time interval of the study, patients were consecutively included without applying any selection criteria. The study protocol was approved by the ethics committee of the University of Heidelberg (Germany, Medical Faculty Heidelberg, S-237/2015). Clinical data comprised basic physiologic and cardiologic parameters (sex, age, weight, BMI, height, smoker), medical history parameters (heart frequency, QT interval and corrected QT interval [QTc] estimated by Bazett’s formula, coronary artery disease degree, ST-elevation myocardial infarction, dilated cardiomyopathy, hypertrophic cardiomyopathy, sleep apnea, hyperlipidemia, hypertension, type 2 diabetes mellitus, catheter ablation), medication (beta blocker, antiarrhythmic drugs, platelet inhibitors, novel oral anticoagulants, vitamin K antagonists, statins, angiotensin receptor blockers, ACE inhibitors, Ca-antagonists, nitrates, diuretics, insulin), and echocardiographic parameters (left ventricular ejection fraction, aortic root diameter, left atrial diameter, interventricular septum diameter, posterior wall diameter, left ventricular end-diastolic diameter, left ventricular end-systolic diameter, inferior vena cava diameter and collapsibility, degree of mitral and tricuspid valve regurgitation, tissue Doppler imaging systolic velocity, early and late diastolic velocity of the mitral annulus, ratio between early diastolic left ventricular filling velocity and passive left ventricular filling velocity, right atrial pressure and systolic pressure of the pulmonary artery). Written informed consent was obtained from all patients, and the study was conducted in accordance with the Declaration of Helsinki. Echocardiographic examinations were carried out because of the following diagnoses: coronary artery disease (25,3%), heart transplantation (5,7%), dilated cardiomyopathy (5,7%), valvular heart disease (5,4%), amyloidosis (5,3%), acute decompensation under an AF episode (4,2%), arrhythmia (2,8%), pulmonary artery hypertension (2,5%), acute inflammatory diseases (1,6%), rheumatic diseases (1,0%), hypertrophic cardiomyopathy (0,3%), chronic obstructive pulmonary disease (0,2%), other diseases (20,5%), or unclear diagnoses (18,7%) as visualized in S1 Fig.

### Echocardiography

Echocardiography examinations were performed on commercially available ultrasound systems (Vivid S5, Vivid i, Vivid 7 and Vivid E9 GE Healthcare Vingmed, Trondheim, Norway and ie33, Philips, Eindhoven, the Netherlands) according to the guidelines of the American Society of Echocardiography [10]. Images included parasternal, apical and subxiphoidal views using 1.5 to 4.0 MHz phase-array transducers. All examinations were performed with 2D echocardiography for anatomic imaging and Doppler echocardiography for assessment of velocities. LA size was determined as the maximal distance between the posterior aortic root wall and the posterior left atrial wall at the end of systole. Aortic root, posterior wall (PW), septum, LV end-systolic diameters (LV, ESD) and end-diastolic diameters (LV, EDD) were obtained in the parasternal long axis view. Inferior vena cava (IVC) diameter was measured in the subxiphoidal view (11). TDI velocities and left ventricular ejection fraction (LV-EF) were measured in the apical four-chamber view. The right atrial pressure (RAP) was estimated from the IVC diameter and its variability during inspiration. If tricuspid valve regurgitation was present, the systolic pressure of the pulmonary artery (sPA) was estimated based on the on the velocity of the tricuspid regurgitant jet and the RAP. Images were digitally stored in a Picture Archiving and Communication System (PACS) and analyzed at clinical workstations (Centricity, GE Healthcare Vingmed, Trondheim, Norway).

### Statistical methods

Continuous variables between SR, pAF and cAF groups were compared using one-way ANOVA. The Bonferroni adjustment was used for multiple post-hoc testing. Standard deviations are indicated by plus-minus signs. Categorical variables between groups were compared with two-tailed Fisher exact test. To predict membership in pAF, cAF and SR groups, we calibrated multivariable logistic models and trained random forest classifiers. Before model calibration, variables were centered by subtracting the arithmetic means. Random forest classifiers containing 200 decision trees, respectively, were trained based on the Adaptive Boosting (AdaBoost) algorithm. For logistic models and random forest classifiers, 100-fold stratified cross-validation was used to determine receiver operating characteristic (ROC) curves and their confidence intervals for pairwise classification between groups. Sequential forward selection was used to find optimal subsets of classification variables. Additional features were selected based on likelihood-ratio testing, assuming that the likelihood-ratio for a model including an additional variable compared to a model without the additional parameter follows a one-dimensional *χ*^2^ distribution. For models obtained by sequential feature selection, we tested if multicollinearity between model variables affected estimation of model coefficients by calculating variance inflation factors (VIFs) for all model variables. VIF values were between 1.01 and 1.46 for variables of the reduced model for classification between pAF and SR, and between 1.12 and 1.38 for variables of the reduced model for classification between cAF and SR, which indicates that influence of multicollinearity for logistic regressions was weak. To assess classification performance, area under the curve (AUC) values, sensitivities, specificities and classification accuracies were analyzed. To transform logistic models to linear scores *L*, logits were scaled between minimal and maximal values for our subject group and in the interval *L* ∈ [0,100]. All analyses were performed based on pre-implemented functions and custom scripts in MATLAB (MathWorks) (see S1 Text for details).

## Results

### Characteristics of the study population

Our study population comprised 1000 patients, 111 patients (11%) with cAF, 161 patients (16%) with pAF and 728 patients (73%) with SR that were examined between 2009 and 2015 at the echocardiography laboratory of the Department of Cardiology at Heidelberg University.

Table 1 gives an overview about demographic and medical history parameters, medication, and echocardiography parameters of the three patient groups. The median patient age at the echocardiographic examination was 61±15 (SR), 68±12 (pAF) and 73±10 (cAF) years, 552 patients were men (55%). Patients with pAF and cAF were of significantly higher age and higher weight than SR patients, and had higher mitral insufficiency and coronary artery disease degrees. Furthermore, pAF and cAF groups were significantly more often affected by hypertension or type 2 diabetes, obtained more often beta blockers, antiarrhythmic drugs, vitamin K antagonists or diuretics, had larger aortic root, LA, IVC or PW diameters, and showed significantly higher systolic TDI velocities of the mitral annulus (S’), lower early diastolic velocities of the mitral annulus (A’), higher RAP or systolic pulmonary artery pressure (sPA) values compared to SR patients. In addition, patients from the cAF group had significantly higher BMI values, had more often sleep apnea, and obtained more often platelet inhibitors, novel oral anticoagulants, Ca-antagonists or insulin than SR patients. Moreover, cAF patients had more often a variable respiratory IVC collapsibility, higher tricuspid insufficiency degrees and higher early diastolic TDI velocities of the mitral annulus (E’) than SR patients. There were fewer differences between pAF and cAF groups. Compared to pAF patients, cAF patients were of significantly higher age, took more often platelet inhibitors, vitamin K antagonists and diuretics, had larger LA diameters, higher early diastolic TDI E’ velocities and higher sPA values.

**Table 1:** Comparison of demographic and clinical parameters between groups.

	SR (n = 728)	pAF (n = 161)	cAF (n = 111)
***Demographics***			
Men, n (%)	384 (53)	102 (63)*	66 (59)
Age (y)	61.1±14.9	68.3±11.7***	72.6±9.7***^††^
Weight (kg)	73.9±14.6	77.6±14.7*	79.4±16.4***
Body mass index (kg/m²)	25.4±4.4	26.1±4.1	27.0±4.8**
Height (cm)	170.4±9.6	172.3±8.8	171.4±9.7
Smokers, n (%)	456 (38)	139 (44)	92 (41)
***Medical history***			
Heart frequency (1/min)	73.0±15.8	77.3±23.6*	75.8±15.4
QT (ms)	392.7±40.4	391.9±56.3	385.6±41.3
QTc (ms)	413.7±31.5	416.5±40.4	411.8±32.9
Coronary artery disease degree	1.2±1.5	1.9±1.6***	2.2±1.7***
STEMI, n (%)	39 (5)	12 (7)	9 (8)
DCM, n (%)	70 (10)	22 (24)	19 (27)
HCM, n (%)	6 (1)	2 (1)	4 (4)
Sleep apnea, n (%)	10 (1)	6 (4)	6 (5)*
Hyperlipidemia, n (%)	358 (49)	105 (65)***	68 (61)
Hypertension, n (%)	457 (62)	123 (76)**	98 (88)***
DM type II, n (%)	133 (18)	47 (29)**	45 (41)***
Catheter ablation, n (%)	5 (1)	5 (3)	2 (2)
***Medication***			
Beta blocker, n (%)	456 (63)	139 (86)***	92 (83)***
Antiarrhytmic, n (%)	33 (5)	60(37)***	57 (51)***
Platelet inhibitor, n (%)	425 (58)	100 (62)	38 (34)***^†††^
NOAC, n (%)	1 (0)	1 (1)	4 (4)**
Vitamin K antagonist, n (%)	82 (11)	74 (46)***	85 (77)***^†††^
Statin, n (%)	402 (55)	85 (53)	59 (53)
ARB, n (%)	167 (23)	36 (22)	27 (24)
ACE inhibitor, n (%)	143 (43)	38 (51)	34 (54)
Ca-antagonist, n (%)	143 (20)	38 (24)	34 (31)*
Nitrate, n (%)	25 (3)	3 (2)	7 (6)
Diuretic, n (%)	32 (42)	14 (59)***	14 (75)***^†^
Insulin, n (%)	32 (4)	14 (9)	14 (13)**
***Echocardiographic parameters***			
LV-EF (%)	50.5±13.3	48.2±13.5	47.4±13.0
Aortic root (mm)	31.6±4.3	33.3±4.3***	32.8±4.7*
LA (mm)	38.5±6.1	43.4±5.9***	47.8±7.0***^†††^
IVS (mm)	11.8±2.7	12.5±2.6*	12.8±2.5**
PW (mm)	11.3±2.1	11.8±2.0*	12.2±1.8***
LV, EDD (mm)	47.8±8.1	48.6±8.0	49.6±7.7
LV, ESD (mm)	33.0±10.0	34.1±10.3	34.8±9.6
IVC (mm)	16.3±3.8	18.3±4.5***	19.5±4.8***
IVC collapsibility, n (%)	673 (92)	139 (86)	85 (77)***
Mitral insufficiency degree	1.6±1.1	1.6±1.0**	1.5±0.8***
Tricuspid insufficiency degree	1.7±1.2	1.6±1.0	1.6±0.8***
TDI, E’ (cm/s)	9.6±3.8	9.4±3.9	10.5±3.2*^†^
TDI, A’ (cm/s)	8.5±3.4	7.1±3.2***	5.0±2.5***^†††^
TDI, E/E’ rest	9.7±6.0	10.3±5.5	10.1±4.9
RAP (mmHg)	5.7±2.3	6.7±3.4***	7.2±3.8***
sPA (mmHg)	30.5±11.5	35.2±12.5***	39.7±13.3***^†^

For continuous parameters and ordinal parameters with more than two levels, means and standard deviations are given, for ordinal parameters with two levels, total counts and percentages are indicated. SR, sinus rhythm; pAF, paroxysmal atrial fibrillation; cAF, chronic atrial fibrillation; QT(c), (corrected) QT interval; coronary artery disease degree with levels 1 to 3 according to the number of affected vessels; STEMI, ST-elevation myocardial infarction; DCM, dilated cardiomyopathy; HCM, hypertrophic cardiomyopathy; DM, diabetes mellitus; NOAC, novel oral anticoagulant; ARB, angiotensin receptor blocker; LV-EF, left ventricular ejection fraction (normal, ≥55%; mild impairment, 45–54%; moderate impairment, 30–44%; severe impairment <30%); LA, left atrium; IVS, interventricular septum; PW, posterior wall; LV, EDD, left ventricular end-diastolic diameter; LV, ESD, left ventricular end-systolic diameter; IVC, inferior vena cava; mitral and tricuspid insufficiency degree with levels 1 to 3 (mild impairment, 1; moderate impairment, 2; severe impairment, 3); TDI, S’, tissue Doppler imaging, systolic velocity of mitral annulus; TDI, E’, tissue Doppler imaging, early diastolic velocity of mitral annulus; TDI, A’, tissue Doppler imaging, late diastolic velocity of mitral annulus; TDI, E/E’, ratio of early diastolic left ventricular filling velocity E and passive left ventricular filling velocity E’; RAP, right atrial pressure; sPA, systolic pressure of the pulmonary artery.

*p<0.05

**p<0.01

***p<0.001 versus SR

^**†**^*p*<0.05

^**††**^*p*<0.01

^**†††**^*p*<0.001 versus pAF from ANOVA followed by Bonferroni multiple comparisons procedure for continuous variables and from Fisher exact test for categorical variables.

Taken together, pAF and cAF groups showed differences to the SR group in similar parameters. In a subset of parameters, only cAF patients differed significantly from SR patients. Interestingly, the parameters age, LA diameter, TDI A’ and sPA, besides vitamin K antagonists or diuretics intake, were significantly different between pAF and SR groups and between cAF and pAF groups, which indicates that pAF pathophysiologically represents an intermediate stage between SR and cAF.

### Echocardiographic parameters allow reliable classification between pAF and SR, and between cAF and SR groups

To calibrate models for classification of patients with unknown AF status between pAF, cAF and SR, we considered all parameters besides the intake of antiarrhythmic drugs, resulting in a total number of 46 variables. For classification between two groups, we pre-selected variables with p-values of *p* < 0.2 for differences between groups. For model calibration, we first included all pre-selected variables. By sequential feature selection, we reduced the logistic models to a smaller set of variables to improve classification performance by avoiding overfitting and to obtain easier manageable model variants. Sequentially adding classification parameters and iteratively testing for a significant log-likelihood improvement resulted in logistic models with 12 variables for the classification between pAF and SR, 8 variables for the classification between cAF and SR (Table 2), or with 3 variables for classification between cAF and pAF (S1 Table).

**Table 2:** Model coefficients and odds ratios for reduced logistic models.

pAF vs. SR				
logistic model with 12 variables				
	coefficient (95% CI)	odds ratio (95% CI)	variable increment	p-value
TDI, A’	-0.1834 (-0.2581, -0.1087)	0.83 (0.77, 0.90)	1 cm/s	1.5·10^−6^
Left atrium	0.4386 (0.2409, 0.6363)	1.55 (1.27, 1.89)	5 mm	1.4·10^−5^
Age	0.3922 (0.2040, 0.5805)	1.48 (1.23, 1.79)	10 years	4.4·10^−5^
Aortic root	0.1020 (0.05244, 0.1507)	1.11 (1.05, 1.16)	1 mm	5.1·10^−5^
Catheter ablation	2.498 (0.9909, 4.004)	12.15 (2.69, 54,83)		0.0012
LV, ESD	-0.1945 (-0.3186, -0.07038)	0.82 (0.73, 0.93)	5 mm	0.0021
Heart rate	0.1783 (0.06284, 0.2939)	1.20 (1.07, 1.34)	10/min	0.0025
Sleep apnea	1.282 (0.1707, 2.393)	3.60 (1.19, 10.94)		0.024
Beta blocker	0.6197 (0.04852, 1.191)	1.86 (1.05, 3.29)		0.033
Hyperlipidemia	0.4872 (0.02126, 0.9532)	1.63 (1.02, 2.59)		0.040
Smoker	0.08833 (-0.1929, 0.3700)	1.09 (0.82, 1.45)		0.54
Diabetes mellitus	-0.02329 (-0.5236, 0.4770)	0.98 (0.59, 1.61)		0.92
Intercept	-2.226 (-2.506, -1.948)			3.3·10^−55^
logistic model with 4 variables				
	coefficient (95% CI)	odds ratio (95% CI)	variable increment	p-value
Age	0.4363 (0.2706, 0.6021)	1.55 (1.31, 1.83)	10 years	2.5·10^−7^
Left atrium	0.4130 (0.2418, 0.5841)	1.51 (1.27, 1.79)	5 mm	2.3·10^−6^
TDI, A’	-0.1382 (-0.2042, -0.07218)	0.87 (0.82, 0.93)	1 cm/s	4.1·10^−5^
Aortic root	0.08023 (0.03476, 0.1257)	1.08 (1.04, 1.13)	1 mm	5.4·10^−4^
Intercept	-2.012 (-2.253, -1,770)			6.9·10^−60^
**cAF vs. SR**				
logistic model with 8 variables				
	coefficient (95% CI)	odds ratio (95% CI)	Variable increment	p-value
Left atrium	1.178 (0.8178, 1.538)	3.25 (2.27, 4.65)	5 mm	1.4·10^−10^
TDI, A’	-0.4700 (-0.6380, -0.3020)	0.63 (0.53, 0.74)	1 cm/s	4.2·10^−8^
Age	0.7695 (0.3875, 1.152)	2.16 (1.47, 3.17)	10 years	7.8·10^−5^
Platelet inhibitor	-1.027 (-1.652, -0.4028)	0.36 (0.19, 0.67)		0.0013
LV, EF	0.2489 (0.09380, 0.4040)	1.28 (1.10, 1.50)	5%	0.0017
QT interval	-1.031 (-1.987, -0.07504)	0.36 (0.14, 0.93)	100 ms	0.035
Beta blocker	1.287 (-0.05311, 2.626)	3.62 (0.95, 13.82)		0.060
Hypertension	0.9705 (-0.05529, 1.996)	2.64 (0.95, 7.36)		0.064
Intercept	-4.940 (-5.854, -4.026)			3.2·10^−26^
logistic model with 3 variables				
	coefficient (95% CI)	odds ratio (95% CI)	Variable increment	p-value
Left atrium	1.040 (0.742, 1.338)	2.83 (2.10, 3.81)	5 mm	8.1·10^−12^
TDI, A’	-0.402 (-0.548, -0.255)	0.67 (0.58, 0.77)	1 cm/s	7.4·10^−8^
Age	0.796 (0.464, 1.129)	2.22 (1.59, 3.09)	10 years	2.7·10^−6^
Intercept	-4.479 (-5.242, -3.716)			1.2·10^−30^

Centered model variables were scaled to representative variable increments as indicated in the fourth column. Coefficients are listed in the order of their importance for classification. CI, confidence interval; TDI, A’, tissue Doppler imaging, velocity during atrial contraction; TDI, E’, tissue Doppler imaging, early diastolic velocity of mitral annulus; LV, ESD, end-systolic left ventricular diameter; LV, EF, left ventricular ejection fraction.

**Fig 1:**
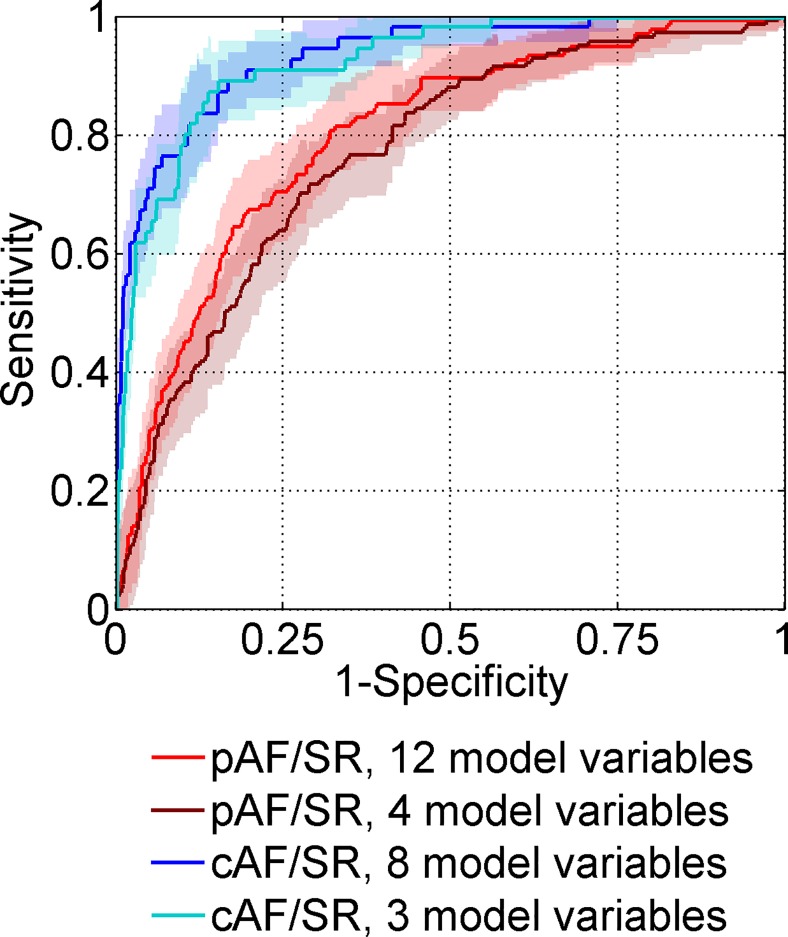
Reliable classification is possible between pAF and SR, and between cAF and SR. ROC curves are plotted for logistic models reduced to the most predictive variables for classification between pAF, cAF and SR groups after 100-fold cross-validation (areas: 95% confidence intervals). AUCs indicate reliable classification between pAF and SR (AUC = 0.80), and between cAF and SR (AUC = 0.93).

Fig 1 shows that logistic models reduced to the most predictive variables allow a reliable classification between pAF and SR, and an even more reliable classification between cAF and SR, which is indicated by large AUC values in ROC curves (pAF/SR, AUC = 0.80; cAF/SR, AUC = 0.93). Compared to logistic models trained on the complete set of pre-selected variables, the reduced models for classification between pAF and SR and between cAF and SR showed higher classification performance (S2 Fig). Contrarily, the reduced model for classification between pAF and cAF showed lower performance than the model trained on the complete set of pre-selected variables (reduced model, AUC = 0.77; full model, AUC = 0.81, S2 Fig). We tested if machine learning techniques could further improve classification performance and trained random forest classifiers. However, these did not show larger AUC values for classification between pAF and SR groups (S3 Fig). For this reason and because of their straightforward interpretability and easy implementation, we decided to focus on logistic models.

Next, we assessed if the reduced logistic models could be further simplified without decreasing their predictive performance. For this reason, we kept only the most significant four (pAF vs. SR) or three variables (cAF vs. SR) with p-values below 10^−4^ (Table 2). AUC values of ROC curves, and specificity as well as classification accuracy percentages at characteristic sensitivity percentages indicate that the simplified model variants yield slightly lower classification performance (Fig 1, Table 3). Classification between cAF and SR was more reliable than between pAF and SR. This is for example indicated by specificity values of about 90% for cAF/SR classification and of about 70% for pAF/SR classification at 80% sensitivity, respectively.

**Table 3:** Classification performance for reduced logistic models.

**pAF vs. SR**		
**logistic model with 12 variables** (TDI A’, left atrium, age, aortic root, catheter ablation, LV ESD, heart rate, sleep apnea, beta blocker, hyperlipidemia, smoker, diabetes mellitus)		
AUC	0.80 (0.76, 0.84)	
Sensitivity	Specificity	Accuracy
70% (61.6%, 78.4%)	76.2% (81.3%, 71.2%)	75.3% (73.2%, 77.3%)
80% (72.6%, 87.4%)	68.1% (73.4%, 62.6%)	70.0% (68.3%, 71.5%)
90% (84.4%, 95.6%)	44.8% (53.0%, 37.0%)	52.0% (50.1%, 54.0%)
**logistic model with 4 variables** (age, left atrium, TDI A’, aortic root)		
AUC	0.77 (0.72, 0.81)	
Sensitivity	Specificity	Accuracy
70% (61.2%, 78.8%)	72.5% (77.8%, 67.1%)	72.1% (70.1%, 74.1%)
80% (73.2%, 86.8%)	58.7% (65.0%, 52.4%)	62.2% (60.1%, 64.2%)
90% (84.5%, 95.5%)	45.0% (53.0%, 37.0%)	52.4% (50.4%, 54.3%)
**cAF vs. SR**		
**logistic model with 8 variables** (left atrium, TDI A’, age, platelet inhibitor, LV EF, QT interval, beta blocker, hypertension)		
AUC	0.93 (0.91, 0.96)	
Sensitivity	Specificity	Accuracy
70% (61.3%, 78.7%)	95.2% (97.4%, 93.0%)	93.5% (92.2%, 94.7%)
80% (71.1%, 88.9%)	89.2% (91.8%, 86.6%)	88.6% (86.5%, 91.0%)
90% (85.2%, 94.8%)	80.4% (84.2%, 76.5%)	81.1% (79.9%, 82.3%)
**logistic model with 3 factors** (left atrium, TDI A’, age)		
AUC	0.92 (0.90, 0.95)	
Sensitivity	Specificity	Accuracy
70% (61.0%, 79.3%)	91.0% (93.6%, 88.5%)	89.6% (87.8%, 91.5%)
80% (70.2%, 89.8%)	89.7% (92.3%, 87.0%)	89.0% (87.0%, 91.0%)
90% (83.2%, 96.7%)	79.2% (83.2%, 75.3%)	80.0% (78.6%, 81.4%)

For reduced logistic models used to classify between pAF and SR or between cAF and SR, specificity and classification accuracy values at 70%, 80% and 90% sensitivity are given. In brackets, 95% confidence intervals are indicated that were estimated by 100-fold cross-validation.

**Fig 2:**
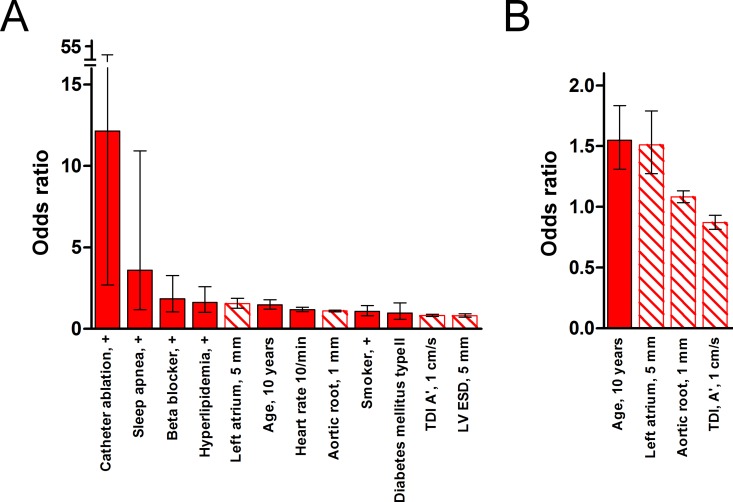
Odds ratios for variables of the model for classification between pAF and SR. (A) Odds ratios for the reduced logistic model with 12 variables ordered according to their magnitude. Odds ratios reflect the effects of binary variable changes, indicated by a ‘+’, or continuous variable changes by the indicated unit intervals, on the risk for the presence of pAF (error bars: 95% confidence intervals, shaded bars: echocardiographic variables). The most predictive variables can be recognized by small confidence intervals. (B) Odds ratios for the simplified logistic model that was reduced to the most predictive 4 variables as in panel A.

In Table 2, estimated model coefficients and *p*-values are provided. Therein, model coefficients are ordered after their importance for classification. Model coefficients were scaled to representative variable increments, which are indicated in the fourth column of Table 2, similar as in the study by Vaziri et al. [7]. Fig 2 and Table 2 show corresponding odds ratios, which equal the values of the exponential function of the model coefficients. The values can be used to assess the effects of continuous variable changes by the indicated variable increments, or of binary variable changes, on the risk for the presence of pAF or cAF. For example, in the model for classification between pAF and SR with 12 variables, an increment of 10 years in age means a risk increase by 1.48 fold for the presence of pAF, while an increment of 5 mm in LA diameter means a risk increase by 1.55 fold.

The binary variables catheter ablation and sleep apnea that were part of the pAF/SR classification model with 12 variables were positive for only a small fraction of the study population, which led to relatively large confidence intervals for model coefficients. Still, the coefficients for these two variables reached significance, which indicates that including the variables was beneficial for the predictive performance of the model. The model for pAF/SR classification further reduced to the four most predictive variables was not dependent on these two variables.

### Linear scores derived from logistic models can serve as decision criterions to initiate further diagnosis for pAF

To obtain an easy implementable decision aid for initializing diagnostic testing for pAF, we transformed the reduced logistic models to linear scores. If a linear score indicates the presence of pAF for a certain parameter set, further diagnostic procedures as a Holter ECG can be applied to validate or disprove the presence of pAF.

To derive linear scores, we transformed the calibrated logistic models with 12 or four variables to linear scores *L*_12_ and *L*_4_. For this purpose, we rescaled the logit values of the logistic models between minimal and maximal values for our subject group and in the interval *L*_4_,*L*_12_ ∈ [0,100] (see S1 Text and S4 Table for details). The score for the model with 12 variables reads

$$\begin{array}{c} L_{12}=48.43+0.3359\cdot \left(\frac{age}{y}-62.39\right)+0.8700\cdot \left(\frac{Ao,root}{mm}-31.92\right)+0.7512\cdot \left(\frac{LA}{mm}-39.37\right)\\ -0.3331\cdot \left(\frac{LV,ESD}{mm}-33.19\right)-1.570\cdot \left(\frac{TDI,A'}{cm/s}-8.285\right)+0.1527\cdot \left(\frac{HF}{1/min}-73.78\right)\\ +10.98\cdot sleep\;apnea+4.172\cdot hyperlipidemia-0.1995\cdot type\;II\;diabetes\\ +0.7565\cdot smoker+5.307\cdot \beta \;Blocker+21.39\cdot Catheter\;ablation\\ =-17,07+0.3359\cdot \frac{age}{y}+0.8700\cdot \frac{Ao,root}{mm}+0.7512\cdot \frac{LA}{mm}-0.3331\cdot \frac{LV,ESD}{mm}-1.570\cdot \frac{DTI,A'}{cm/s}\\ +0.1527\cdot \frac{HF}{1/min}+10.98\cdot sleep\;apnea+4.172\cdot hyperlipidemia-0.1995\cdot type\;II\;diabetes\\ +0.7565\cdot smoker+5.307\cdot \beta \;Blocker+21.39\cdot Catheter\;ablation \end{array}.$$ (1)

In the first line of Eq 1, the average variable values for our subject group are subtracted from each continuous variable, which was simplified to the second line. In Eq 1, continuous variables are divided by their units (y, years; mm, millimeters; cm/s, centimeters per second; 1/min, per minute) to obtain dimensionless contributions to the score. To test with 80% sensitivity, a score of *L*_12_ ≥ 58.35 predicts the presence of pAF. For example, a 65 years old patient with an aortic root diameter of 35 mm, an LA diameter of 39 mm, an LV ESD of 32 mm, a TDI A’ velocity of 12 cm/s and a heart frequency of 67/min, smoker, without sleep apnea, type 2 diabetes or catheter ablation but with hyperlipidemia and beta blocker intake will have a score value of *L*_12_ = 55.48. In this case, the model predicts that the patient has SR. If the LA diameter of the patient, however, was 43 mm or larger, the presence of pAF is predicted, which suggests conducting further validating diagnostic investigations. Accordingly, the score for the simplified model with four variables reads

$$\begin{array}{c} L_{4}=61.67+0.4997\cdot \left(\frac{age}{y}-62.39\right)+0.9188\cdot \left(\frac{Ao,root}{mm}-31.92\right)\\ +0.9459\cdot \left(\frac{LA}{mm}-39.37\right)-1.583\cdot \left(\frac{TDI,A'}{cm/s}-8.285\right)\\ =-22.96+0.4997\cdot \frac{age}{y}+0.9188\cdot \frac{Ao,root}{mm}\\ +0.9459\cdot \frac{LA}{mm}-1.583\cdot \frac{TDI,A'}{cm/s} \end{array}.$$ (2)

**Fig 3:**
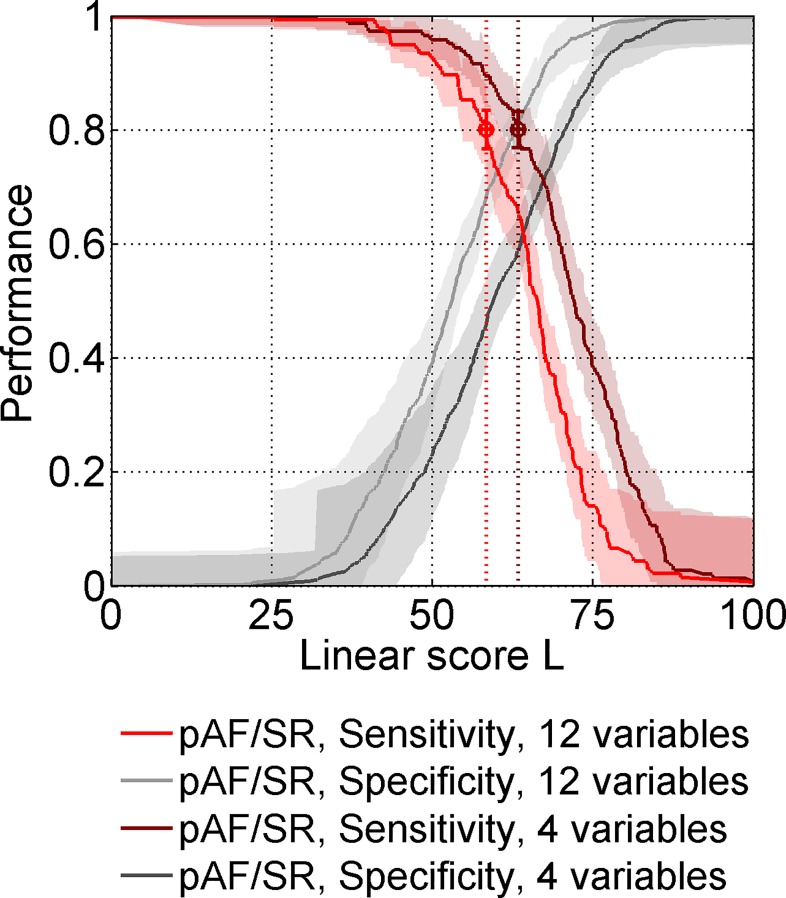
Classification performance of linear model scores. Sensitivities (red) and specificities (grey) are indicated for different values of the pAF/SR classification scores with 12 or 4 parameters. Threshold score values for classification with 80% sensitivity are indicated (L_12_ = 58.35 for the model with 12 variables and L_4_ = 63.32 for the model with 4 variables, error bars: standard deviations, shaded areas: 95% confidence intervals).

As in Eq 1, variables are divided by their units to obtain dimensionless contributions. If a value of *L*_4_ ≥ 63.32 is exceeded, which represents the threshold score at 80% sensitivity, a patient will be predicted to have pAF. Fig 3 shows how classification sensitivity and specificity depend on the model scores that are chosen as classification thresholds. To facilitate application of the scores with 12 or 4 parameters, we included a score calculator in S4 Table.

Because the fraction of pAF patients is distinctively smaller than the fraction of SR patients, despite high sensitivity and specificity, the precision of the classification will be moderate. Given the constitution of our study sample, which represents an unbiased sample of patients attending our echocardiographic laboratory, at 80% classification sensitivity, a precision of 36% will result. This means that for patients, which either have SR or pAF, about 64% of the patients with a positive classification result will be false positives.

## Discussion

We studied the predictive power of a combination of echocardiographic and additional clinical parameters in order to develop a predictive score for pAF in patients undergoing an echocardiography examination. In our set of predictive variables, random forest classifiers showed no improvement to logistic models, which are preferable because of their easy applicability. By sequential feature selection, we obtained reduced models with 12 variables for pAF/SR classification and eight variables for cAF/SR classification. A further simplified model for classification between pAF and SR with the four variables age, LA diameter, TDI A’ and aortic root diameter showed ROC curves with a slightly lower AUC value compared to the 12 parameter model.

It was previously described by several studies that AF patients show LA enlargement [7,11]. A study by Sanfillipo et al. found that atrial enlargement can occur as a consequence of AF and concludes that maintenance of SR may prevent atrial enlargement [12]. Accordingly, the developed models predict that in patients undergoing an echocardiographic investigation, an LA diameter increase by 5 mm increases the risk for pAF by approximately 1.5 fold (Table 2). In contrast to these well-established empirical findings, the origin of the relation between LA enlargement and AF are speculative and topic of molecular research.

TDI A’ velocity was characterized as a predictor of atrial function in several previous studies, reviewed in [13]. In AF, TDI A’ is reduced due to an impaired atrial relaxation [14]. Therefore, it is physiologically reasonable that this parameter is involved in the development of pAF. Here, our models for pAF vs. SR classification predict that an increase by 1 cm/s is accompanied by an about 0.8 fold decrease of pAF risk. Further model parameters were aortic root and left ventricular end-systolic diameters, which stand in a causal relation to hemodynamic consequences of atrial remodeling for ventricular function. The associated atrial electrical remodeling can lead to an increased heart frequency, which causes hemodynamic changes in the atria [15,16]. Age is known as an important risk factor of AF [4,5,17]. The molecular mechanism of myocardial aging is not well understood and is a topic of basic research [18]. The strong influence of age in the development of AF is reflected in the model scores, which predict that an increment by 10 years increases the risk for pAF by about 1.4 fold. Furthermore, the score variables sleep apnea, smoker, hyperlipidemia and type 2 diabetes mellitus are known risk factors of AF [4,19]. That the variable beta blocker was included in the score can be explained by an association between AF and coronary artery disease, which was significantly more frequent in AF patients (Table 1).

Similar to our study, Mathew et al. developed a model score to predict occurrence of AF subsequent to coronary arterial bypass grafting surgery, which was based on clinical parameters and parameters related to the surgical procedure [17]. As in our models, the study by Mathew et al. found that age and beta blocker intake were predictive for AF. However, this study did not distinguish between pAF and cAF.

The transition from SR to pAF and finally to cAF is caused by structural, electrical and contractile atrial remodeling, which is reflected by changes in physiological properties of the myocardium [4,5]. Here, we found that especially the echocardiographic model parameters LA diameter and TDI A’, besides the patient age, which significantly differed between SR and pAF and between pAF and cAF groups, are descriptive for this transition process. Taken together, these findings are consistent with the actual understanding of AF pathology.

## Limitations

We concentrated on parameters that were routinely documented in our echocardiographic laboratory. A limitation of this study is that the patient population was restricted to only one cardiology center and the score was only retrospectively tested. Echocardiographic investigations were carried out by different physicians. Subsequently to the developments of predictive scores in this study, it will be possible to perform a prospective evaluation of the scores in patients undergoing an echocardiographic investigation.

## Conclusion

In conclusion, we developed a logistic model based on 47 echocardiographic and other clinical parameters from routine cardiac assessment to predict the presence of pAF. Datasets from 1000 patients were included to obtain high statistical significance. We learned that logistic regression models allowed higher predictive power for pAF prediction compared to a common machine learning procedure. Patients with pAF and cAF showed significant differences to SR patients in a similar set of diagnostic parameters. Especially, echocardiographic measures were highly predictive for the presence of AF, compared to other clinical parameters. The four most predictive variables for classification between pAF and SR were TDI A’, LA diameter, age and aortic root diameter. A logistic model for discrimination between pAF and SR shows an AUC value of 0.80, which resembles a classifier with high predictive power. For an easy implementation, logistic models were transformed to linear scores. We think that the developed model scores are furthermore valuable to describe the pathophysiological process of AF-associated atrial remodeling on a quantitative basis. Taken together, the developed model scores represent a simple, non-invasive tool for detecting pAF that can be easily implemented to clinical practice and might serve as a new decision aid to initiate further diagnostic investigations for validating the presence of pAF.

## Supporting Information

S1 Fig Composition of the study group. (PDF)

S2 Fig Reduction of logistic models affects classification performance. (PDF)

S3 Fig Comparison between random forest classifiers and logistic regression models. (PDF)

S4 Fig Logistic model scores and derived linear scores. (PDF)

S1 Table Model coefficients, odds ratios and classification performance of cAF vs. pAF classification model. (PDF)

S2 Table Model coefficients and odds ratios for logistic models reduced to significant parameters. (PDF)

S3 Table Classification performance of logistic models reduced to significant parameters. (PDF)

S4 Table Score calculator. (XLSX)

S1 Text Transformation of logistic models to linear scores. (PDF)
