# Supplementary material for: A Simple, Non-Invasive Score to Predict Paroxysmal Atrial Fibrillation
Source: PLoS One. 2016 Sep 28;11(9):e0163621. doi: 10.1371/journal.pone.0163621 (PMC5040399; doi:10.1371/journal.pone.0163621)
Supplement: S1 Fig — (PDF) [file pone.0163621.s001.pdf]

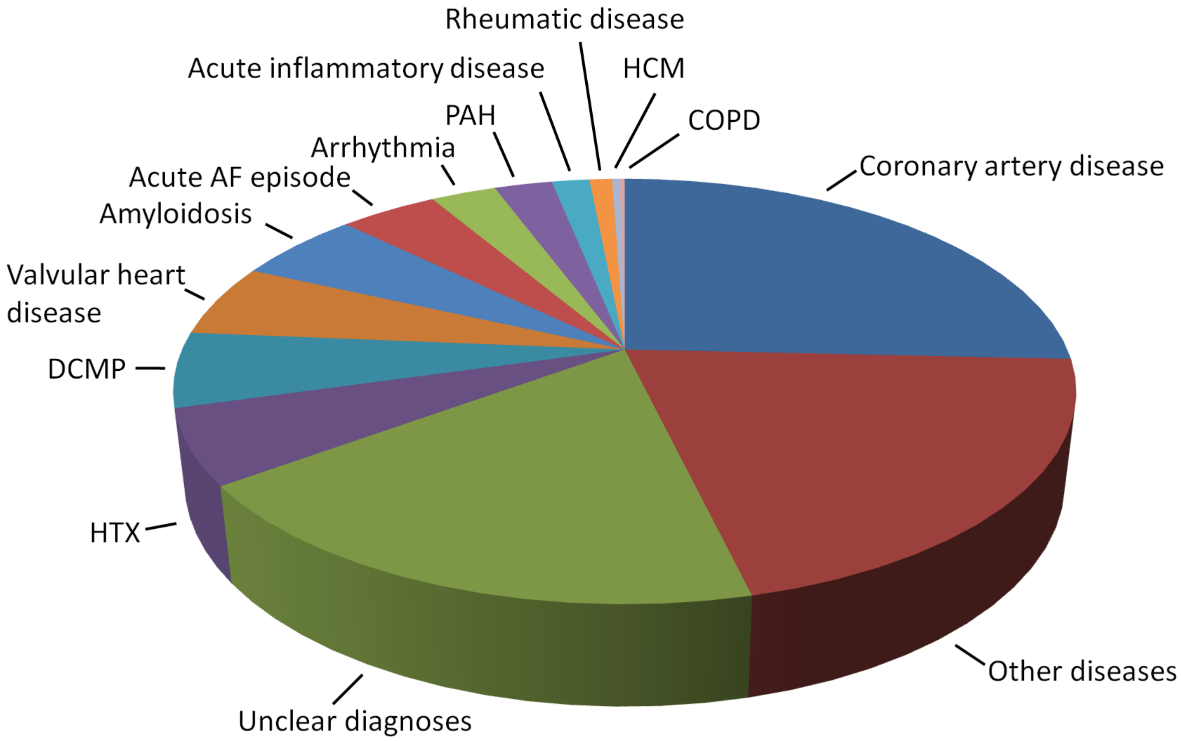

Fig S1. Composition of the study group. Indication for an echocardiographic examination were given because of coronary artery disease (25.3%), heart transplantation (HTX, 5.7%), dilated cardiomyopathy (DCMP, 5.7%), valvular heart disease (5.4%), amyloidosis (5.3%), acute decompensation under an AF episode (4.2%), arrhythmia (2.8%), pulmonary artery hypertension (PAH, 2.5%), acute inflammatory diseases (1.6%), rheumatic diseases (1.0%), HCM, hypertrophic cardiomyopathy (0.3%), chronic obstructive pulmonary disease (COPD, 0.2%), other diseases (20.5%), or unclear diagnoses (18.7%).
