# Supplementary material for: A Simple, Non-Invasive Score to Predict Paroxysmal Atrial Fibrillation
Source: PLoS One. 2016 Sep 28;11(9):e0163621. doi: 10.1371/journal.pone.0163621 (PMC5040399; doi:10.1371/journal.pone.0163621)
Supplement: S2 Fig — (PDF) [file pone.0163621.s002.pdf]

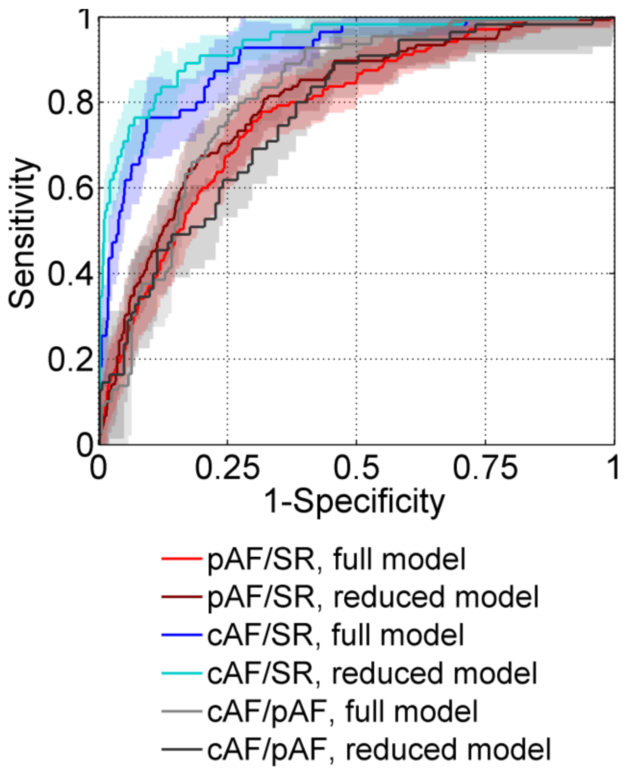

Fig S2. Reduction of logistic models affects classification performance. By sequential feature selection, full logistic models for classification between pAF, cAF and SR groups that contained all pre-selected variables were systematically reduced to variables, which significantly contributed to classification. The ROC curves, obtained from 100-fold cross-validation, indicate an improved classification performance for the reduced model for classification between pAF and SR (full model: AUC=0.78, reduced model with 12 variables: AUC=0.80), an improved classification performance of the reduced model for classification between cAF and SR (full model: AUC=0.90, reduced model with eight variables: AUC=0.93), and a decreased classification performance of the reduced model for classification between pAF and cAF (full model: AUC=0.81, reduced model with three variables: AUC=0.77, areas: 95% confidence intervals).
