# Supplementary material for: A Simple, Non-Invasive Score to Predict Paroxysmal Atrial Fibrillation
Source: PLoS One. 2016 Sep 28;11(9):e0163621. doi: 10.1371/journal.pone.0163621 (PMC5040399; doi:10.1371/journal.pone.0163621)
Supplement: S3 Fig — (PDF) [file pone.0163621.s003.pdf]

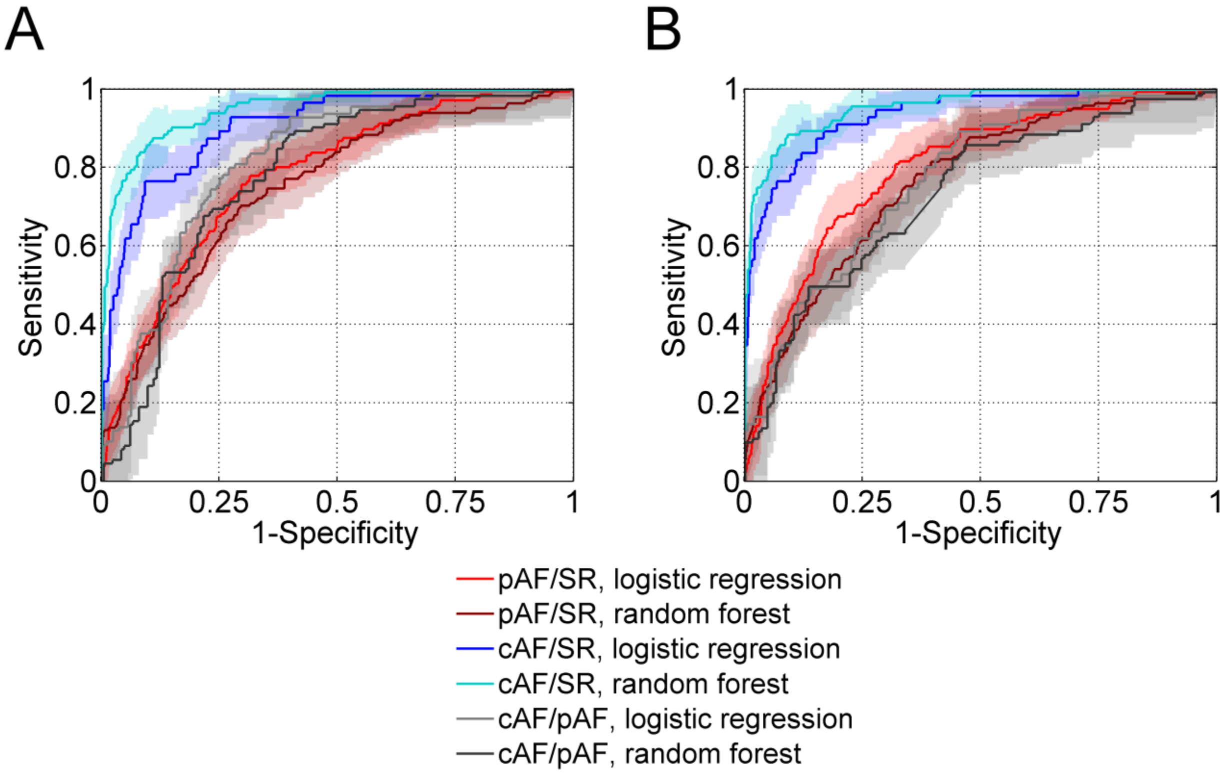

Fig S3. Comparison between random forest classifiers and logistic regression models. (A) ROC curves for logistic regression models and random forest classifiers were obtained by 100-fold cross-validation based on the full set of variables for classification between pAF and SR (logistic regression: AUC=0.78, random forest classification: AUC=0.75), cAF and SR (l. r.: AUC=0.90, r. f. c.: AUC=0.95), and between cAF and pAF (l. r.: AUC=0.81, r. f. c.: AUC=0.78, areas: 95% confidence intervals). (B) ROC curves for reduced models obtained by sequential feature selection for classification between pAF and SR (l. r.: AUC=0.80, random forest classification: AUC=0.76), cAF and SR (l. r.: AUC=0.93, r. f. c.: AUC=0.95), and between cAF and pAF (l. r.: AUC=0.77, r. f. c.: AUC=0.74) as in panel A.
