# Supplementary material for: A Simple, Non-Invasive Score to Predict Paroxysmal Atrial Fibrillation
Source: PLoS One. 2016 Sep 28;11(9):e0163621. doi: 10.1371/journal.pone.0163621 (PMC5040399; doi:10.1371/journal.pone.0163621)
Supplement: S4 Fig — (PDF) [file pone.0163621.s004.pdf]

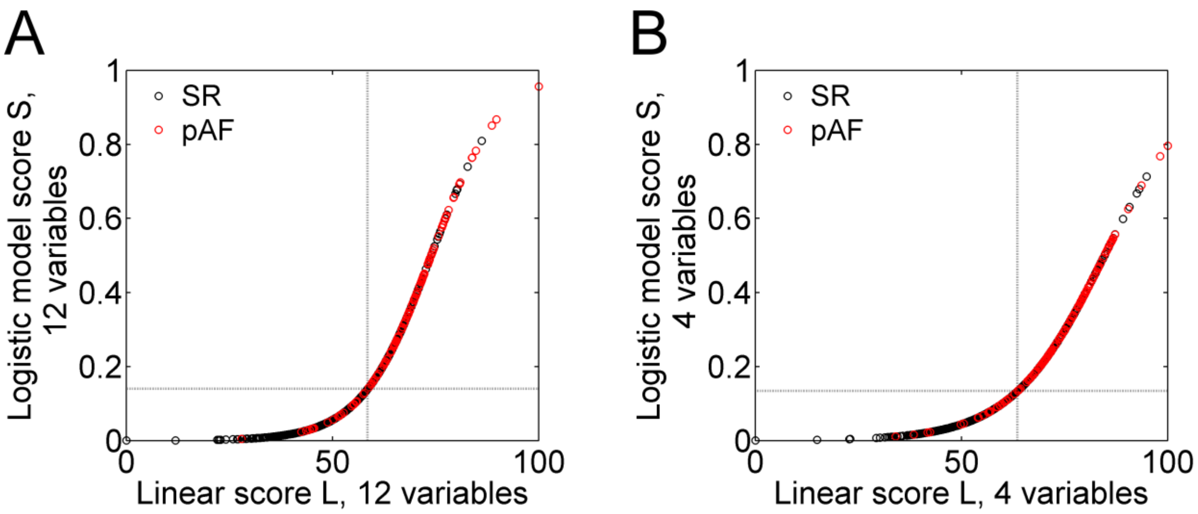

Fig S4. Logistic model scores and derived linear scores. (A) Score values of the logistic model with 12 variables and corresponding linear score values for all SR (black circles) and pAF patients (red circles). Dotted lines indicate threshold scores at 80% classification sensitivity ($S_{12} = 0.1394, L_{12} = 58.35$), predicted based on 100-fold cross-validation. (B) Score values of the logistic model with 4 variables, corresponding linear score values, and threshold scores at 80% classification sensitivity ($S_4 = 0.1337, L_4 = 63.32$) as in panel A.
