# Supplementary material for: A Simple, Non-Invasive Score to Predict Paroxysmal Atrial Fibrillation
Source: PLoS One. 2016 Sep 28;11(9):e0163621. doi: 10.1371/journal.pone.0163621 (PMC5040399; doi:10.1371/journal.pone.0163621)
Supplement: S1 Table — (PDF) [file pone.0163621.s005.pdf]

S1 Table. Model coefficients, odds ratios and classification performance of cAF vs. pAF classification model.

pAF vs. cAF, model coefficients				
	coefficient (95% CI)	odds ratio (95% CI)	variable increment	p-value
Left atrium	0.6555 (0.3483, 0.9627)	1.93 (1.42, 2.62)	5 mm	$2.9 \cdot 10^{-5}$
TDI, A’	-0.2443 (-0.3948, -0.09378)	0.78 (0.67, 0.91)	1 cm/s	0.0015
TDI, E’	0.1107 (0.006404, 0.2149)	1.12 (1.01, 1.24)	1 cm/s	0.037
Intercept	-1.138 (-1.534, -0.7429)			$1.7 \cdot 10^{-8}$

pAF vs. cAF, classification performance		
AUC	0.77 (0.67, 0.88)	
Sensitivity	Specificity	Accuracy
70% (61.8%, 78.2%)	67.4% (74.1%, 60.6%)	68.4% (62.4%, 74.4%)
80% (72.0%, 87.9%)	61.7% (69.3%, 54.1%)	66.8% (60.6%, 73.1%)
90% (83.4%, 96.6%)	49.6% (59.3%, 40.0%)	61.2% (56.1%, 66.3%)

Upper part: Centered model variables were scaled to representative variable increments as indicated in the fourth column. Coefficients are listed in the order of their importance for classification. *Lower part:* Specificity and classification accuracy values at 70%, 80% and 90% sensitivity are given. In brackets, 95% confidence intervals are indicated that were estimated by 100-fold cross-validation. CI, confidence interval; TDI, A’, tissue Doppler imaging, velocity during atrial contraction; TDI, E’, tissue Doppler imaging, early diastolic velocity of mitral annulus.
