# Supplementary material for: A Simple, Non-Invasive Score to Predict Paroxysmal Atrial Fibrillation
Source: PLoS One. 2016 Sep 28;11(9):e0163621. doi: 10.1371/journal.pone.0163621 (PMC5040399; doi:10.1371/journal.pone.0163621)
Supplement: S2 Table — (PDF) [file pone.0163621.s006.pdf]

S2 Table. Model coefficients and odds ratios for logistic models reduced to significant parameters.

	coefficient (95% CI)	odds ratio (95% CI)	variable increment	p-value
**pAF vs. SR**				
logistic model with 10 variables				
TDI, A'	-0.1654 (-0.2368, -0.09393)	0.85 (0.79, 0.91)	1cm/s	$5.7 \cdot 10^{-6}$
Left atrium	0.4378 (0.2458, 0.6298)	1.55 (1.28, 1.88)	5 mm	$7.9 \cdot 10^{-6}$
Age	0.3633 (0.1821, 0.5446)	1.44 (1.20, 1.72)	10 years	$8.6 \cdot 10^{-5}$
Aortic root	0.09488 (0.04729, 0.1425)	1.10 (1.05, 1.15)	1 mm	$9.3 \cdot 10^{-5}$
Catheter ablation	2.375 (0.8789, 3.871)	10.75 (2.41, 48.0)		0.0019
LV, ESD	-0.1787 (-0.2973, -0.0602)	0.84 (0.74, 0.94)	5mm	0.0031
Heart rate	0.1552 (0.04243, 0.2681)	1.17 (1.04, 1,31)	10/min	0.0070
Beta blocker	0.6729 (0.1109, 1.235)	1.96 (1.12, 3.44)		0.019
Sleep apnea	1.187 (0.0869, 2.287)	3.28 (1.09, 9.85)		0.034
Hyperlipidemia	0.4753 (0.03083, 0.9198)	1.61 (1.03, 2.51)		0.036
Intercept	-2.153 (-2.421, -1.886)			$3.8 \cdot 10^{-56}$

	coefficient (95% CI)	odds ratio (95% CI)	variable increment	p-value
**cAF vs. SR**				
logistic model with 6 variables				
Left atrium	1.244 (0.8940, 1.595)	3.47 (2.44, 4.93)	5 mm	$3.4 \cdot 10^{-12}$
TDI, A'	-0.4660 (-0.6277, -0.3043)	0.63 (0.53, 0.74)	1 cm/s	$1.6 \cdot 10^{-8}$
Age	0.8660 (0.5049, 1.227)	2.38 (1.66, 3.41)	10 years	$2.6 \cdot 10^{-6}$
LV, EF	0.2270 (0.07721, 0.3768)	1.25 (1.08, 1.46)	5 %	0.0030
Platelet inhibitor	-0.8691 (-1.469, -0.2695)	0.42 (0.23, 0.76)		0.0045
QT interval	-1.011 (-1.941, -0.0808)	0.36 (0.14, 0.92)	100 ms	0.033
Intercept	-4.682 (-5.503, -3.861)			$5.6 \cdot 10^{-29}$

Centered model variables were scaled to representative variable increments as indicated in the fourth column. Coefficients are listed in the order of their importance for classification. CI, confidence interval; LV, ESD, end-systolic left ventricular diameter; LV, EF, left ventricular ejection fraction; TDI, A', tissue Doppler imaging, velocity during atrial contraction.
