# Supplementary material for: A Simple, Non-Invasive Score to Predict Paroxysmal Atrial Fibrillation
Source: PLoS One. 2016 Sep 28;11(9):e0163621. doi: 10.1371/journal.pone.0163621 (PMC5040399; doi:10.1371/journal.pone.0163621)
Supplement: S3 Table — (PDF) [file pone.0163621.s007.pdf]

S3 Table. Classification performance of logistic models reduced to significant parameters.

**pAF vs. SR**<br>logistic model with 10 variables<br>(age, aortic root, left atrium, LV ESD, sleep apnea, hyperlipidemia, beta blocker, catheter ablation, TDI A’, heart rate)		
AUC	0.79 (0.75, 0.84)	
Sensitivity	Specificity	Accuracy
70% (62.0%, 78.1%)	73.5% (78.7%, 68.3%)	73.0% (71.0%, 75.0%)
80% (72.7%, 87.3%)	64.4% (70.1%, 58.8%)	67.0% (65.2%, 68.7%)
90% (84.5%, 95.5%)	48.0% (55.2%, 40.0%)	54.5% (52.9%, 56.1%)

**cAF vs. SR**<br>logistic model with 6 variables<br>(age, left atrium, LV EF, platelet inhibitor, TDI A’, QT interval)		
AUC	0.93 (0.91, 0.96)	
Sensitivity	Specificity	Accuracy
70% (61.5%, 79.0%)	94.2% (96.5%, 92.0%)	93.0% (91.1%, 94.0%)
80% (71.3%, 88.7%)	92.0% (94.3%, 92.0%)	91.0% (89.4%, 92.7%)
90% (84.2%, 95.8%)	80.3% (84.1%, 76.4%)	81.0% (79.8%, 82.3%)

For logistic models that were reduced to significant parameters, used to classify between pAF and SR or between cAF and SR, specificity and classification accuracy values at 70%, 80% and 90% sensitivity are given. In brackets, 95% confidence intervals are indicated that were estimated by 100-fold cross-validation.
