# Supplementary material for: A Simple, Non-Invasive Score to Predict Paroxysmal Atrial Fibrillation
Source: PLoS One. 2016 Sep 28;11(9):e0163621. doi: 10.1371/journal.pone.0163621 (PMC5040399; doi:10.1371/journal.pone.0163621)
Supplement: S1 Text — (PDF) [file pone.0163621.s009.pdf]

S1 Text. Development of logistic models and transformation to linear scores.

To fit multivariate logistic models, we used the MATLAB function *glmfit* with option *binomial*. The sequential feature selection algorithm consists of stepwise including further model variables and testing if model performance is improved (sequential forward selection). We made use of the sequential feature selection algorithm by applying the MATLAB function *sequentialfs*. For cross-validation, datasets were split into training and test datasets using the function *cvpartition*. ROC curves and AUC values were determined using the function *perfcurve*. Random forest classifiers were calibrated using the *fitensemble* function.

Based on the estimated logistic regression parameters, we constructed linear scores that can serve as easy implementable decision aid to initialize further diagnostic validation of the presence of pAF.

The logistic model with 12 variables x_1 to x_{12}, rescaled by the arithmetic means $\bar{x}_1$ to $\bar{x}_{12}$, contains the intercept value α_0 and coefficients α_1 to α_{12}

$$S_{12} = \frac{\exp(\alpha_0 + \alpha_1(x_1 - \bar{x}_1) + \ldots + \alpha_{12}(x_{12} - \bar{x}_{12}))}{1 + \exp(\alpha_0 + \alpha_1(x_1 - \bar{x}_1) + \ldots + \alpha_{12}(x_{12} - \bar{x}_{12}))} = \frac{1}{\exp(-\alpha_0 - \alpha_1(x_1 - \bar{x}_1) - \ldots - \alpha_{12}(x_{12} - \bar{x}_{12})) + 1}.$$

In Table 2, we scaled coefficients obtained from logistic regression for illustrative reasons to representative variable increments. With coefficients that were not rescaled, which can be obtained by dividing coefficient values in the first column of Table 2 by increments in the fourth column of Table 2, the equation for the logistic model score with 12 variables reads

$$S_{12} = \frac{1}{\exp\left(\begin{array}{c} 2.227 - 0.03922\cdot\left(\frac{\text{age}}{\text{y}} - 62.39\right) - 0.1016\cdot\left(\frac{\text{Ao, root}}{\text{mm}} - 31.92\right) - 0.08772\cdot\left(\frac{\text{LA}}{\text{mm}} - 39.37\right) \\ + 0.03890\cdot\left(\frac{\text{LV, ESD}}{\text{mm}} - 33.19\right) + 0.1834\cdot\left(\frac{\text{TDI,A'}}{\text{cm/s}} - 8.285\right) - 0.01783\cdot\left(\frac{\text{HF}}{\text{1/min}} - 73.78\right) \\ - 1.282\cdot(\text{sleep apnea} - 0.01800) - 0.4872\cdot(\text{hyperlipidemia} - 0.5214) + 0.02329\cdot(\text{type II diabetes} - 0.2027) \\ - 0.08833\cdot(\text{smoker} - 0.3991) - 0.6197\cdot(\beta\ \text{Blocker} - 0.6693) - 2.498\cdot(\text{Catheter ablation} - 0.01125) \end{array}\right) + 1}$$

(Eq. S1)

Therein, *Ao root* stands for aortic root diameter, *LA* for the LA diameter, *LV, ESD* for the end-systolic left ventricular diameter, *TDI,A'* for the tissue Doppler imaging velocity during atrial contraction, and *HF* for heart frequency. In Equation S1, continuous variables are divided by their units (y, years; mm, millimeters; cm/s, centimeters per second; 1/min, per minute) to obtain dimensionless contributions to the score. To test with 80% sensitivity, the score has to be $S_{12} \geq 0.1394$ for classification as pAF, which is indicated in S4 Fig. Analogously, the simplified logistic model score for classification between pAF and SR with 4 variables is given by the equation

$$S_4 = \frac{1}{\exp\left(\begin{array}{c} 2.012 - 0.04363 \cdot \left(\frac{\text{age}}{\text{y}} - 62.39 \right) - 0.0802 \cdot \left(\frac{\text{Ao,root}}{\text{mm}} - 31.92 \right) \\ - 0.08260 \cdot \left(\frac{\text{LA}}{\text{mm}} - 39.37 \right) + 0.1382 \cdot \left(\frac{\text{TDI,A'}}{\text{cm/s}} - 8.285 \right) \end{array} \right) + 1}$$

(Eq. S2)

As in Equation S1, variables are divided by their units. Again, the coefficient values in Equation S2 equal the scaled coefficients in the first column of Table 2 divided by increments in the fourth column of Table 2. For testing with 80% sensitivity, a threshold score of $S_4 \geq 0.1338$ as indicated in S4 Fig has to be attained for classification as pAF.

To construct linear scores L_{12} and L_4, we scaled the logits of Equations S1 and S2 between the minimal and maximal values for all included patients and in the interval $L_4, L_{12} \in [0,100]$. Accordingly, values of 0 or 100 represent the minimal or maximal scores for our subject group. With

$$f_{12}(x_1,...,x_{12}) = \alpha_0 + \alpha_1(x_1 - \bar{x}_1) + ... + \alpha_{12}(x_{12} - \bar{x}_{12})$$

the linear score with 12 variables reads

$$L_{12} = \frac{100}{\max(f_{12}) - \min(f_{12})} \left(\alpha_0 + \alpha_1(x_1 - \bar{x}_1) + ... + \alpha_{12}(x_{12} - \bar{x}_{12}) - \min(f_{12}) \right). \quad \text{(Eq. S3)}$$

Inserting the rescaled coefficients, $\min(f_{12}) = -8.633$ and $\max(f_{12}) = 3.044$, in Eq. S3 and simplifying the equation results in Eq. 1. Similarly, Eq. 2 was obtained by inserting the rescaled coefficients for the model with 4 variables, $\min(f_4) = -7.397$ and $\max(f_4) = 1.335$ in and according equation for L_4. In S4 Fig, we illustrate classification by the logistic models (Equations S1 and S2) and the derived linear scores (Equations 1 and 2).

Besides models for classification between pAF and SR with 12 or 4 variables and models for classification between cAF and SR, we additionally evaluated the performance of model versions of intermediate size (S2 Table and S3 Table). In these model versions, we eliminated parameters with p-values p>0.05, resulting in a model with 10 parameters for classification between pAF and SR, and a model with 6 parameters for classification between cAF and SR. The model for classification between pAF and SR showed slightly decreased performance, compared to the version with 12 parameters. We therefore focused on the version with 12 parameters and the significantly simplified version with 4 parameters.
